# Evaluation of epigenetic age calculators between preeclampsia and normotensive pregnancies in an Australian cohort

**DOI:** 10.1038/s41598-022-05744-4

**Published:** 2022-01-31

**Authors:** Paulina Pruszkowska-Przybylska, Shaun Brennecke, Eric K. Moses, Phillip E. Melton

**Affiliations:** 1 Department of Anthropology, Faculty of Biology and Environmental Protection, University of Łódź, 90-237 Łódź, Poland; 2 Pregnancy Research Centre, Department of Maternal‐Fetal Medicine, Royal Women’s Hospital, Melbourne, VIC Australia; 3 Department of Obstetrics and Gynaecology, The Royal Women’s Hospital, The University of Melbourne, Melbourne, VIC Australia; 4 Menzies Institute for Medical Research, University of Tasmania, Hobart, TAS Australia; 5 School of Global Population Health, University of Western Australia, Perth, Australia; 6 School of Biomedical Science, University of Western Australia, Perth, Australia

**Keywords:** DNA, Genetics research, Epigenetics, Hypertension

## Abstract

Advanced biological aging, as assessed through DNA methylation markers, is associated with several complex diseases. The associations between maternal DNA methylation age and preeclampsia (PE) have not been fully assessed. The aim of this study was to examine if increased maternal DNA methylation age (*DNAm*Age) was shown to be accelerated in women with PE when compared to women who had normotensive pregnancies. The case/control cohort available for study consisted of 166 women (89 with normotensive pregnancy, 77 with PE) recruited previously at the Royal Women’s Hospital in Melbourne, Australia. DNA methylation profiles were obtained using the Illumina EPIC Infinium array for analysis of genomic DNA isolated from whole blood. These profiles were used to calculate seven estimates of *DNAm*Age and included (1) Horvath, (2) Hannum, (3) Horvath Skin and Blood, (4) Wu, (5) PhenoAge, (6) telomere length and (7) GrimAge and its surrogate measures. Three measures of DNA methylation age acceleration were calculated for all seven measures using linear regression. Pearson's correlation was performed to investigate associations between chronological age and *DNAm*Age. Differences between chronological age and *DNAm*Age and epigenetic age acceleration were investigated using t-tests. No significant difference was observed for chronological age between women with PE (age = 30.53 ± 5.68) and women who had normotensive pregnancies (age = 31.76 ± 4.76). All seven *DNAm*Age measures were significantly correlated (*p* < 0.001) with chronological age. After accounting for multiple testing and investigating differences in *DNAm*Age between normotensive women and women with PE, only Wu *DNAm*Age was significant (*p* = 0.001). When examining differences for epigenetic age acceleration between PE and normotensive women Hannum, Wu, and PhenoAge *DNAm*Age estimates (*p* < 0.001) were significant for both epigenetic age acceleration and intrinsic acceleration models. We found that accelerated maternal *DNAm*Age is increased in women with PE in some models of epigenetic aging. This research underlines the importance for further investigation into the potential changes of differential DNA methylation in PE.

## Introduction

Preeclampsia (PE) is a complex pregnancy specific disorder clinically characterized by new onset hypertension and proteinuria affecting 3—5% of pregnant women^1^. Known risk factors for PE include first pregnancy, obesity, multiple gestation, periodontal diseases, advanced maternal age, inadequate diet, and family history^2,3^. Family and population studies have shown that PE has a large heritable component^4–8^. While there has been some success in the identification of maternal susceptibility genes for PE^9–11^ they do not explain all the heritability. This indicates that other genomic factors, including epigenetic modifications, may be involved in predisposing some women to risk of developing PE.

Studies on the epigenetic modification of DNA methylation across time and different tissues has led to the development of ‘epigenetic clocks’ as potential biomarkers for several complex diseases and disorders^12^. These composite DNA methylation measures of age (*DNAm*Age) estimate an individual’s biological age over several DNA methylation loci (CpGs) from blood or other biological tissues. Those individuals with an estimated *DNAm*Age above their chronological age are then considered to be aging at an accelerated rate. The regression of *DNAm*Age on chronological age is defined as epigenetic age acceleration and has been shown to be an accurate predictor of several complex disease including cancer^13^, cardiovascular disease^14^, and all-cause and cause-specific mortality^15^.

Several *DNAm*Age measures have been developed that differ based on their composition of CpGs and outcome (Table 1). These *DNAm*Age measures include Horvath^16^, Hannum^17^, Horvath Skin and Blood^18^, and Wu^19^ and are widely utilised due to their robust ability to correlate with chronological age or biological age discrepancies in various tissues. However, as these *DNAm*Age measures were initially developed with chronological age as their primary outcome, they do not detect those individual DNA methylation differences associated with biological decline beyond that of advancing age. This has led to the more recent development of *DNAm*Age measures for mortality prediction that incorporate composite outcomes of age-related clinical measurements that differentiate between healthy and unhealthy aging. These include PhenoAge^20^ and GrimAge^21^, which have been developed using longitudinal data and well-defined mortality outcomes, thus providing the ability to provide potential useful biomarkers of biological age. Finally, telomere length (TL), which is known to be associated with biological aging and complex disease has also been shown to be estimated from a subset of CpGs. This estimated measure of TL using DNA methylation markers has been shown to be more strongly correlated with chronological age and complex disease than TL as traditionally measured from leukocytes^22^.

**Table 1:** Characteristics of the methyl clocks.

DNAm- age estimators	Number of included CpGs	Type of the cell/tissue	Remarks
**Chronological age predictors**			
Horvath^16^	335 CpGs	Sorted cell types, tissues, and organs	Eighteen of the original 353 CpGs were not included in our analyses as they are not available on the EPIC array
Hannum^17^	71 CpGs	Immune blood types to age by weighting with cytotoxic T cells, exhausted cytotoxic T cells, and plasmablasts	It was measured using 65 of the original 71 CpGs
Horvath Skin and Blood^18^	391 CpGs	Skin and blood cells	None
Wu^19^	111 cpG	Blood cells	More precise estimator in the case of younger individuals
**Mortality predictors**			
PhenoAge^20^	513 CpGs	Blood cell composition	None
GrimAge^21^	1030 CpGs	Blood samples	The composition of 8 DNA methylation-based biomarkers for plasma proteins and self-reported smoking based on packs per year. The plasma protein surrogates include: cystatin C, leptin, tissue inhibitor metalloproteinases 1 (TIMP1), adrenomedullin (ADM), beta-2-microglobulin (B2M), growth differentiation factor 15 (GDF15), and plasminogen activation inhibitor 1 (PAI-1)
**Telomere length**			
TL^22^	140 CpGs	Blood cells	None

Previous research of *DNAm*Age and PE has demonstrated that differential DNA methylation may be an indicator of the accelerated placental aging observed in early onset PE pregnancies^23,24^. While advanced maternal age has been shown to be associated with increased risk for PE^25,26^, differences in *DNAm*Age measures have not been widely investigated in maternal DNA methylation profiles. A study by Heisenberg et al.^27^ did not identify any significant differences between DNA methylation age and PE in a multi-ethnic cohort. However, their investigation only included a single *DNAm*Age measure and did not investigate difference in any of the more recent *DNAm*Age mortality predictors.

The purpose of this study was to evaluate different *DNAm*Age measures to determine if significant differences could be identified between women who had been diagnosed with PE and those who had normotensive pregnancies. We hypothesize that using composite epigenetic scores such as those employed in *DNAm*Age measures may be a useful tool for understanding PE susceptibility. To address the relationship between chronological age and *DNAm*Age we evaluated seven different *DNAm*Age measures to determine if an association could be identified between normotensive and preeclamptic women in a pregnancy cohort of Australian women^28^.

## Material and methods

### Study population

**Figure 1:**
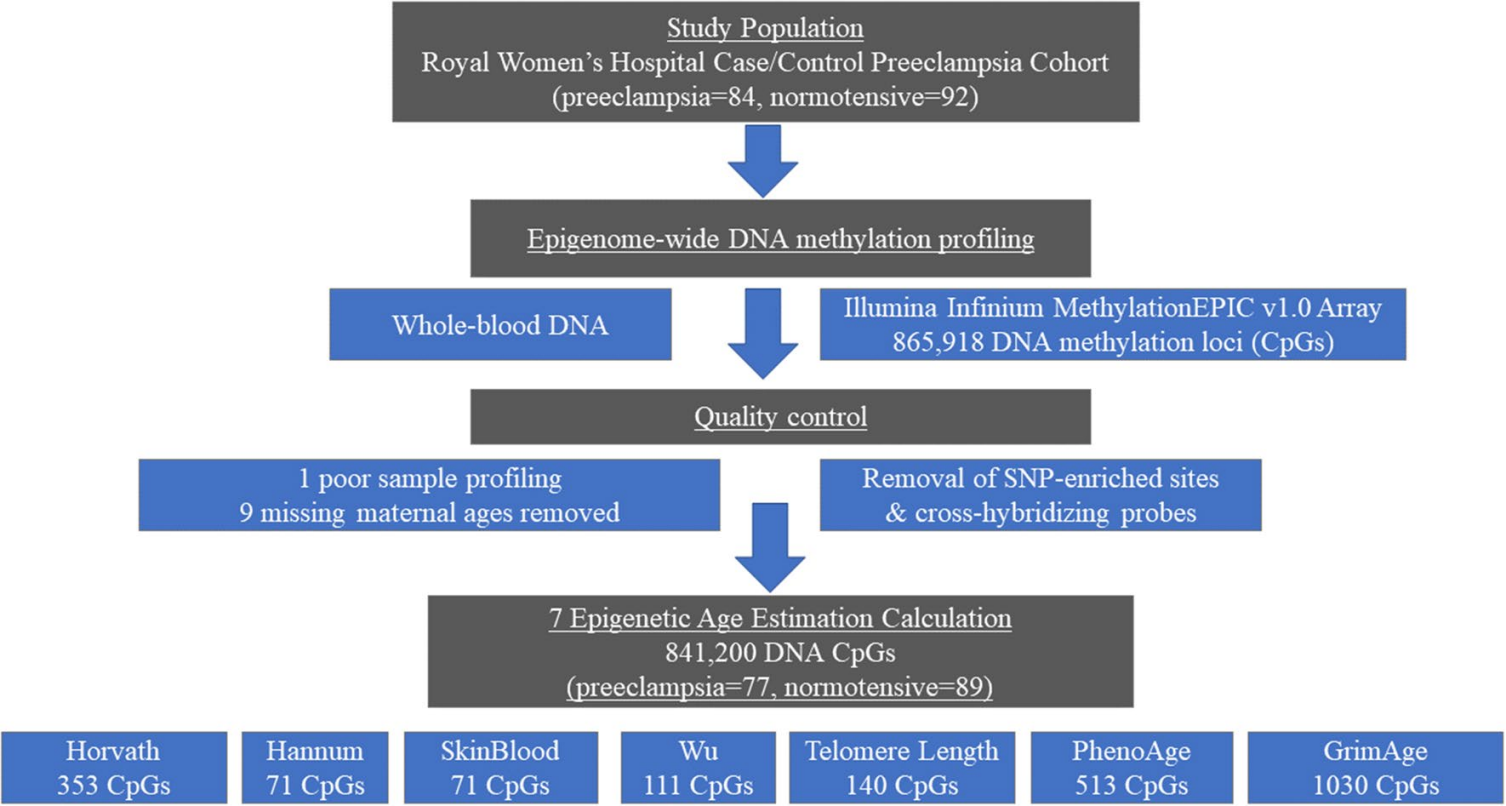
Schematic showing workflow for the study.

The Australian case–control cohort of 166 unrelated women used in this study included 77 PE cases and 89 normotensive pregnancy controls randomly and retrospectively ascertained from a larger Australian case–control cohort of 1,774 women that were recruited at the Royal Women's Hospital (RWH), Melbourne, Australia over a five period from 2007 to 2011. The Australian population seen at the RWH in Melbourne is ∼70% Caucasian and for this current study the focus was on the inclusion of subjects of confirmed European genetic ancestry (Fig. 1). Blood samples were collected at the end of pregnancy after the diagnosis of PE or normality was made.

### Preeclampsia diagnosis

PE diagnosis was determined by qualified clinicians using criteria set by the Australasian Society for the Study of Hypertension in Pregnancy^29,30^ and the Society of Obstetric Medicine of Australia and New Zealand for the management of hypertensive diseases of pregnancy^31^. Women were considered to have PE if they were previously normotensive and if they, on at least two occasions six or more hours apart, had after 20 weeks gestation (i) a rise in systolic blood pressure (SBP) of at least 25 mmHg and/or a rise from baseline diastolic blood pressure (DBP) of at least 15 mmHg, or (ii) SBP ≥ 140 mmHg and/or DBP ≥ 90 mmHg. Additionally, significant new onset proteinuric levels were either ≥ 0.3 g/l in a 24-h specimen, at least a ‘2 + ’ proteinuria dipstick reading from a random urine collection or a spot protein∶creatine ratio ≥ 0.03 g/mmol. Women with PE who also experienced convulsions or unconsciousness in their perinatal period were classified as having eclampsia. Women with pre-existing hypertension or other medical conditions known to predispose for PE (e.g. renal disease, diabetes, twin pregnancies or fetal chromosomal abnormalities) were excluded. Of the 1,774 unrelated Australian women initially recruited for this study, 1,018 women were of confirmed Caucasian ancestry, meeting our inclusion criteria. Of these, 471 were confirmed, by medical records, as having PE (cases) and 547 were confirmed as having a normal pregnancy (controls). Of the 166 women participants for this study, 77 women were confirmed, as having PE and 89 women were confirmed as having a normotensive pregnancy.

### DNA extraction and epigenome-wide DNA methylation profiling

This study utilized existing genomic DNA (gDNA) samples that had been extracted from whole blood using Qiagen's Blood and Cell Culture DNA Midi Kit (Qiagen Pty Ltd, Doncaster, VIC, Australia). The 176 participant gDNA samples were bisulfite converted and DNA methylation profiled using the Illumina EPIC Infinium (Illumina, San Diego, CA, United States) array, by Pathwest Laboratory Medicine (Perth, Western Australia). The R package *RnBeads*^32^ was used to perform quality control of the epigenome-wide DNA methylation profiles and to perform sex checks. After removing SNP-enriched sites and probes with a high likelihood of cross-hybridization one gDNA sample and CpGs with the highest fraction of unreliable measurement were removed. A total of 175 samples and 841,200 DNA methylation probes were then normalized using the Beta-Mixture Quantile dilation (BMIQ) model^33^. Maternal age data was not available for 9 samples, leaving 166 samples for epigenetic age calculation (PE = 77, normotensive = 89).

### Cell-type heterogeneity and DNAmAge calculation

To account for cell type heterogeneity, we used the Houseman-based reference method^34^ and included the estimated proportion of neutrophils, monocytes, basophils, natural killer cells, CD4 + T and CD8 + T cells for each participant sample. In addition, we calculated the cell type heterogeneity values CD8pCD28nCD45Ran, CD8 Naïve, and PlasmaBlast used by Horvath to estimate intrinsic epigenetic age acceleration (IEAA).

Six *DNAm*Age estimates (Horvath, Hannum, Horvath Skin and Blood, Wu, PhenoAge, and TL) were calculated using BMIQ normalized DNA methylation β-values in the R package*, methylclock*^35^. In addition, we calculated GrimAge as previously described^21^. GrimAge is calculated as the composite of 8 DNA methylation-based biomarkers for plasma proteins and self-reported smoking based on packs per year. The plasma protein surrogates include cystatin C, leptin, tissue inhibitor metalloproteinases 1 (TIMP1), adrenomedullin (ADM), beta-2-microglobulin (B2M), growth differentiation factor 15 (GDF15), and plasminogen activation inhibitor 1 (PAI-1). The selection and calculation of these surrogates from DNA methylation array data, along with their function and disease association have been previously described^18^. Each of the surrogates is denoted by the presence of its surrogate with the prefix “*DNAm*”, i.e., *DNAm*GDF15 for the surrogate growth differentiation factor 15. The number of CpGs markers utilised for each *DNAm*Age estimator utilised are shown in Fig. 1. As not all CpGs are available across different Illumina epigenome DNA methylation arrays, missing CpG values were imputed.

For six (excluding TL) of these seven *DNAm*Age measures we calculated three biomarkers of epigenetic age acceleration. These included: *the difference between chronological age and DNAm*Age *(ΔDNAm*Age*)*, which represents the difference between DNA methylation and chronological ages; *epigenetic age acceleration* (EAA), which represents the residuals from using linear regression to regress chronological age on each epigenetic age measure, and *intrinsic epigenetic age acceleration* (IEAA)*,* which represents the residuals from a multivariable linear regression where chronological age was regressed on each *DNAm*Age measure adjusted for estimated cell type heterogeneity. The difference between EAA and IEAA are that IEAA captures cellular age acceleration independently of blood cell proportions known to vary across the lifespan.

### Statistical analysis

All statistical analyses were performed in R version 3.6.1^36^. Standard descriptive statistics were computed, and data were examined graphically and statistically for missingness, outliers, and normality. Each of the 7 *DNAm*Age estimates was compared with chronological age using Pearson's correlation and examined visually using scatterplots. All group differences in age-adjusted age metrics were investigated using an independent sample unpaired two tail T-test for equal variance between participants diagnosed with PE and those with a normotensive pregnancy. To account for multiple testing, we used a Bonferroni correction (0.05/7) of *p* < 0.007 as our threshold for statistical significance.

### Ethics approval

This study was conducted according to the guidelines of the Declaration of Helsinki, and all procedures involving research study participants were approved by the University of Western Australia Human Research Ethics Committee, the Royal Women's Hospital Research and Ethics Committees, Melbourne, Australia, and the Institutional Review Board of the University of Texas Health Science Center at San Antonio, San Antonio, Texas, USA. All study participants gave their written informed consent when enrolled in the study.

## Results

The average chronological age of women with normotensive pregnancies was 31.76 ± 4.96 and for those with PE was 30.53 ± 5.68 (Table 2). Seven *DNAm*Age measures were evaluated. For four of these (Horvath, Hannum, PhenoAge, and GrimAge), *DNAm*Age estimates were higher than chronological age for both groups of women. For two, *DNAm*Age measures (Horvath skin and blood and Wu), estimates were lower than chronological age for both groups. For TL, where the average TL in the general population is 8^22^, values were lower in both groups.

**Table 2:** Pearson's correlations and independent t-tests between average DNAm_age estimators among investigated women.

DNAMAGE estimators	Normotensive (*N* = 89)				Preeclamptic (*N* = 77)						
	Mean	Std.Dev	r	p	Mean	Std.Dev	r	*p*	t	*p*	Cohen’s D
chronological age	31.764	4.755	-	–	30.533	5.679	–	–	− 1.521	0.130	0.050
**Chronological age predictors**											
Horvath	36.110	5.544	0.648	< 0.001	36.273	6.775	0.597	< 0.001	0.170	0.865	2.630
Hannum	34.792	4.641	0.652	< 0.001	36.573	5.249	0.705	< 0.001	2.320	0.022	0.360
Horvath skin and blood	29.762	5.456	0.765	< 0.001	30.092	6.656	0.721	< 0.001	0.351	0.726	< 0.01
Wu	9.920	0.808	0.265	0.012	10.328	0.806	0.627	< 0.001	3.242	0.001	< 0.01
**Mortality predictors**											
PhenoAge	33.111	6.374	0.609	< 0.001	35.866	7.715	0.529	< 0.001	2.519	0.013	1.450
GrimAge	39.510	5.371	0.660	< 0.001	39.594	5.231	0.632	< 0.001	− 0.101	0.919	6.720
**Telomere length**											
TL	7.495	0.160	− 0.484	< 0.001	7.446	0.163	− 0.503	< 0.001	-1.951	0.053	< 0.01

### Pearson correlation of chronological and DNAmAge measures

Pearson correlations were calculated between seven *DNAm*Age measures (Horvath, Hannum age, PhenoAge, Horvath skin and blood, Wu, GrimAge, and TL) and chronological age in women with normotensive pregnancies and PE. Six *DNAm*Age measures were significantly (*p* < 0.007) positively correlated with chronological age (Table 2). The strongest correlation was for Horvath skin and blood (r = 0.721; *p* < 0.001 for PE and r = 0.765; *p* < 0.001 for normotensive women) and the weakest for PhenoAge (r = 0.529; *p* < 0.001 for PE and r = 0.609; *p* < 0.001 for normotensive women). Only TL was significantly negatively correlated with chronological age (r =  − 0.503; *p* < 0.001 for PE and r =  − 0.484; *p* < 0.001 for normotensive women).

### Independent t-tests between normotensive and PE

**Figure 2:**
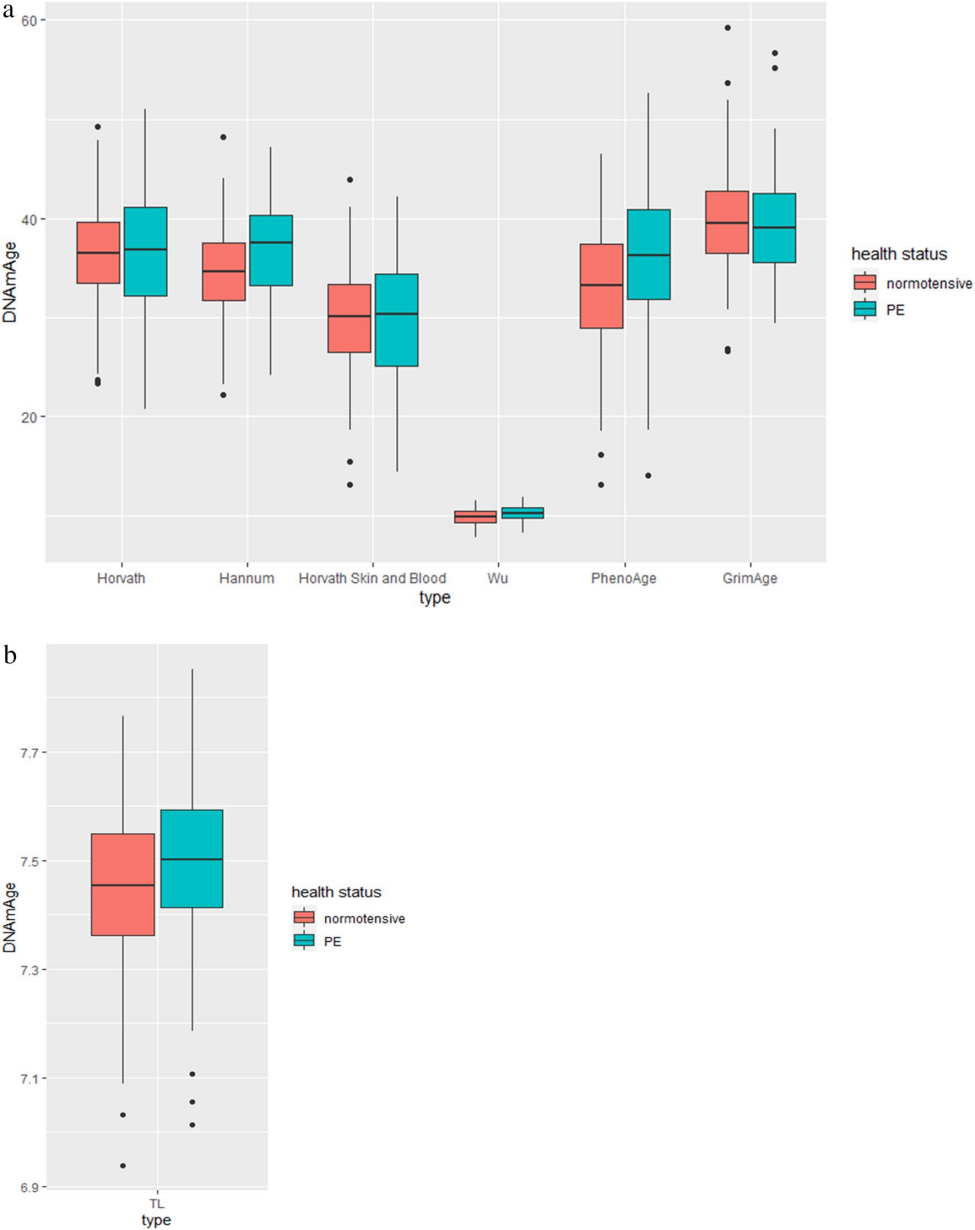
(**a**) Chronological age compared with six different *DNAm*Age models between women with normotensive and preeclamptic (PE) pregnancies. (**b**). Measure of TL DNAmAge between women with normotensive and preeclamptic (PE) pregnancies.

Independent unpaired two-tail t-tests with unequal variance were used to test for differences between *DNAm*Age and chronological age for women with normotensive pregnancies and those with PE (detailed in Table 2 and Fig. 2a, b). After accounting for multiple testing, the only *DNAm*Age measure that was statistically significantly different (*p* < 0.001) between normotensive women and those with severe PE was Wu. Both PhenoAge (*p* < 0.013) and Hannum (*p* < 0.02) were nominally associated with differences between PE and normotensive women.

### Comparisons of epigenetic age acceleration between normotensive and PE

**Figure 3:**
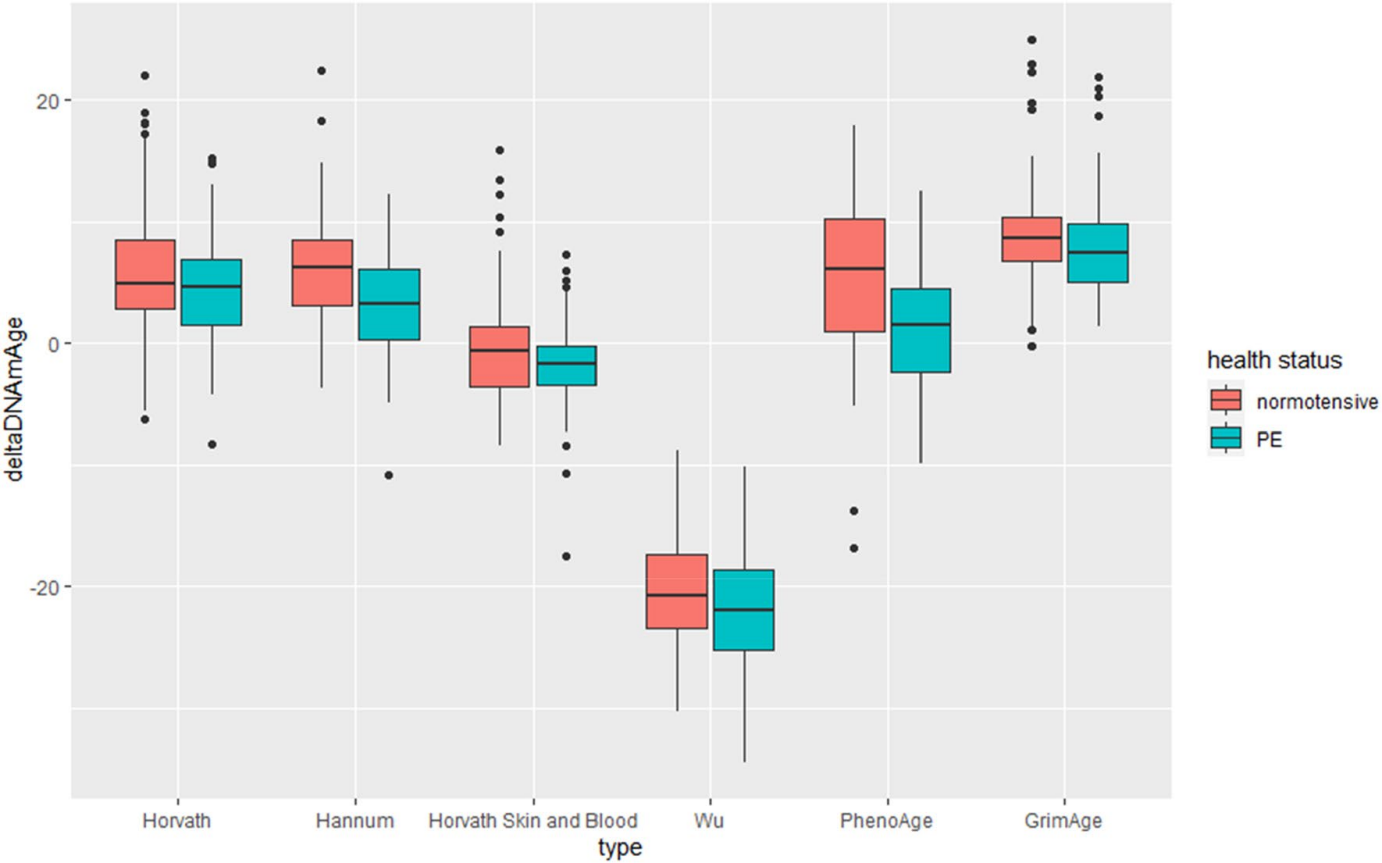
Six different Δ*DNAm*Age which represents the absolute difference between *DNAm*Age and chronological age in women with normotensive and preeclamptic (PE) pregnancies.

**Figure 4:**
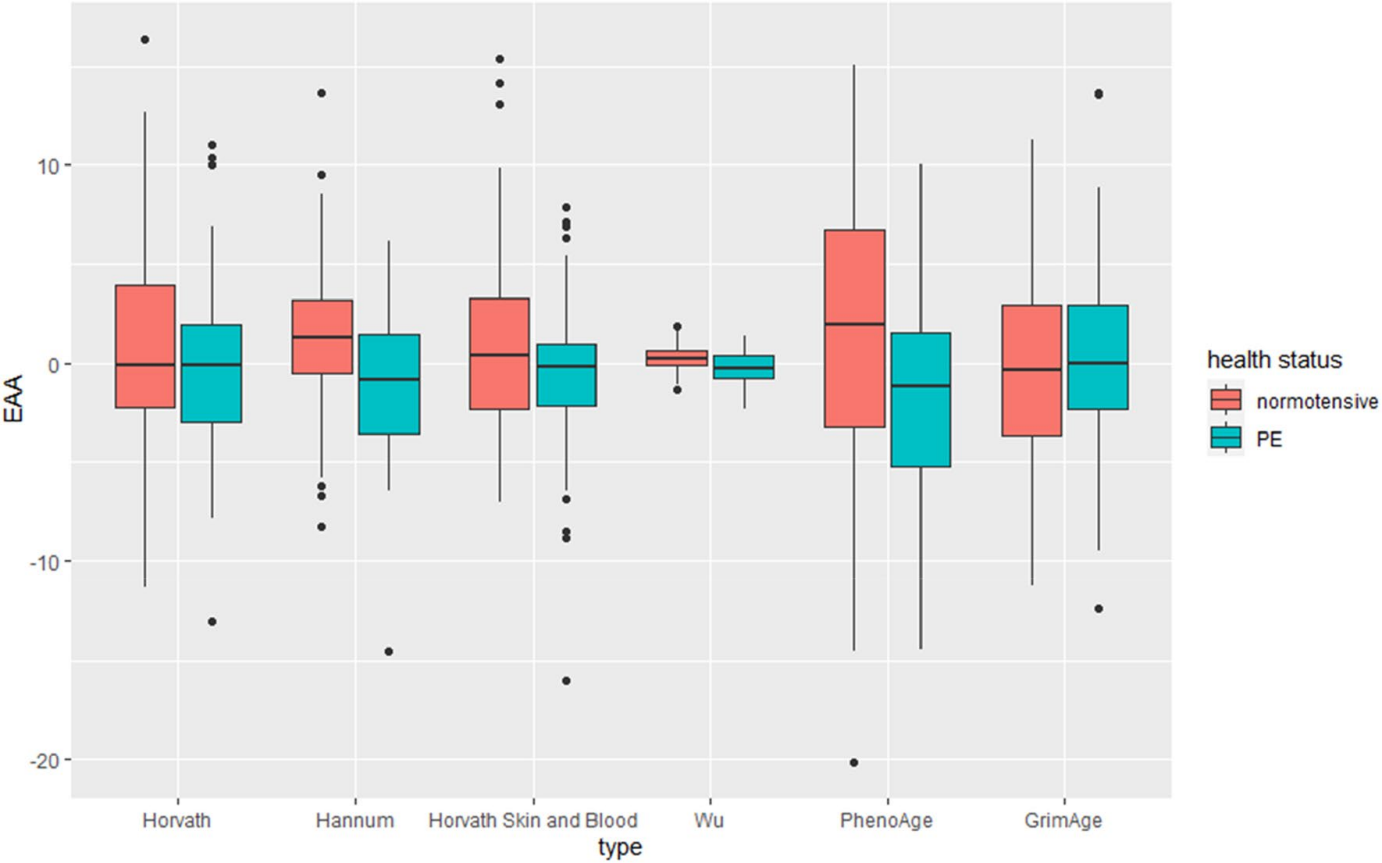
Epigenetic age acceleration (EAA), which represents the residuals from a linear regression model of chronological age with six different *DNAm*Age measurse.

**Figure 5:**
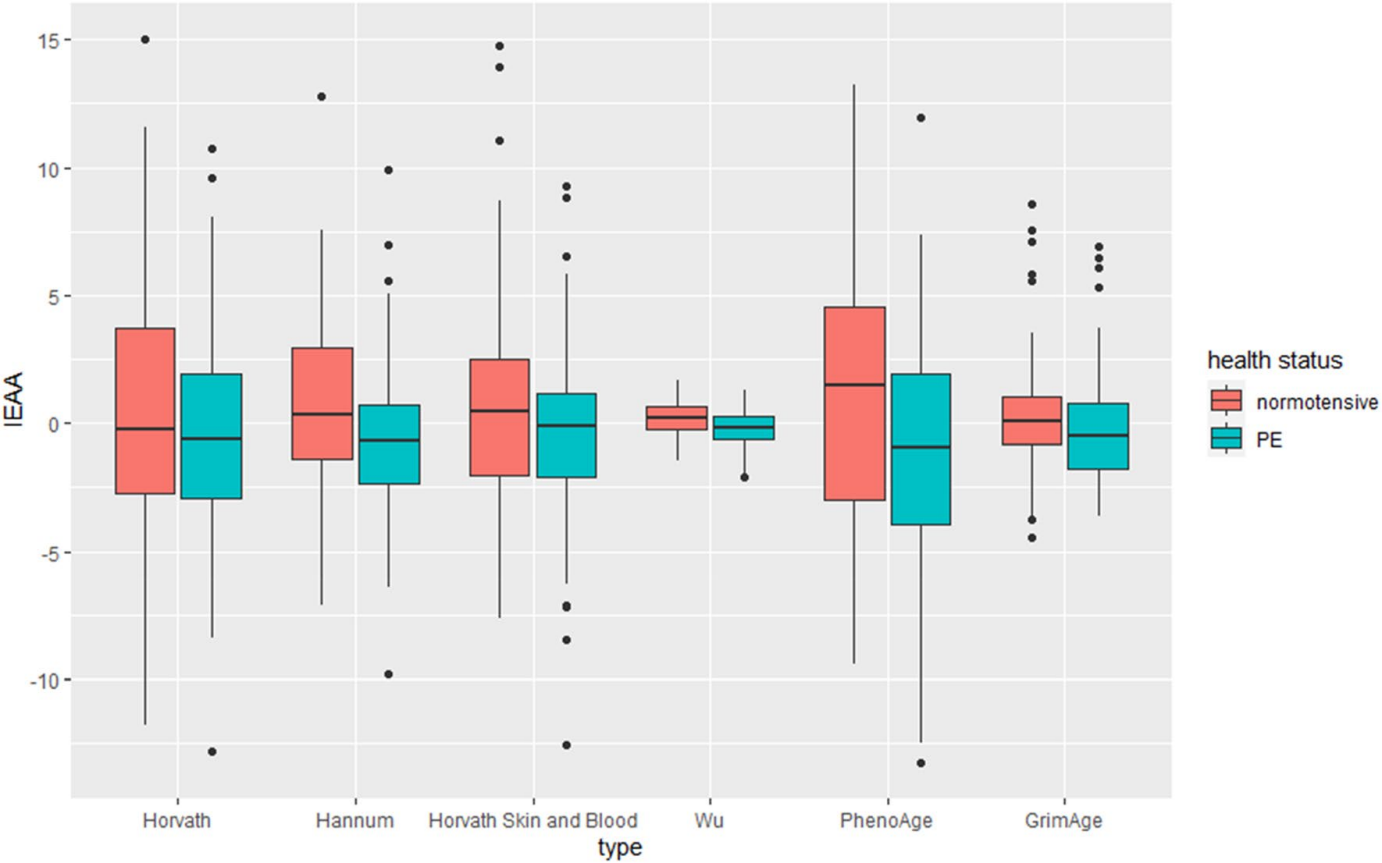
Intrinsic epigenetic age acceleration (IEAA), which represents the residuals from regressing chronological age on each DNAmAge measure adjusted for estimated cell type.

Differences between measures of epigenetic age acceleration among women with normotensive pregnancies and those with PE are shown in Table 3 and Figs. 3, 4, 5. After accounting for multiple testing *ΔDNAm*Age was shown to be significantly accelerated (*p* < 0.001) for women with PE than those women with normotensive pregnancies for Hannum and PhenoAge. EAA was significantly (*p* < 0.001) accelerated in PE women compared to normotensive women in the Hannum, Levine and Wu models. For IEAA the *DNAm*Ages Hannum, Levine and Wu were significantly accelerated (< 0.001) in women with PE when compared to those who had normotensive pregnancies.

**Table 3:** Independent t-tests between average accelerated mDNA age estimators among investigated women: ΔDNAmAge – the difference between DNA methylation and chronological age, EAA- Epigenetic Age Acceration IEAA—the intrinsic epigenetic age acceleration.

DNAm_age estimators	Normotensive (*N* = 89)		Preeclamptic (*N* = 77)		t	*p*	Cohen’s D
	Mean	Std.Dev	Mean	Std.Dev			
**ΔDNAmAge**							
Chronological age predictors							
Horvath	4.346	4.377	5.740	5.678	1.784	0.076	0.280
Hannum	3.028	3.919	6.040	4.218	4.767	< 0.001	0.740
Horvath Skin and Blood	− 2.003	3.560	− 0.441	4.696	2.433	0.016	< 0.01
Wu	− 21.844	4.607	− 20.205	5.212	2.151	0.033	< 0.01
Mortality predictors							
PhenoAge	1.347	5.132	5.334	6.739	4.319	< 0.001	0.010
GrimAge	8.978	4.751	7.830	4.143	1.663	0.098	0.40
**Epigenetic age acceration (EAA)**							
Chronological age predictors							
Horvath	− 0.487	4.224	0.562	5.438	1.397	0.164	0.220
Hannum	− 1.178	3.518	1.361	3.730	4.509	< 0.001	0.010
Horvath Skin and Blood	− 0.635	3.516	0.735	4.613	2.167	0.032	< 0.01
Wu	− 0.226	0.784	0.261	0.645	4.323	< 0.001	< 0.01
Mortality predictors							
PhenoAge	− 1.690	5.078	1.954	6.547	4.033	< 0.001	0.010
GrimAge	− 0.362	4.996	0.313	4.542	− 0.911	0.364	0.070
**Intrinsic epigenetic age acceration (IEAA)**							
Chronological age predictors							
Horvath	− 0.398	3.994	0.460	5.196	1.202	0.231	1.850
Hannum	− 0.770	3.051	0.891	3.338	3.348	0.001	< 0.01
Horvath Skin and Blood	− 0.485	3.300	0.560	4.517	1.717	0.088	< 0.01
Wu	− 0.186	0.728	0.215	0.666	3.687	< 0.001	< 0.01
Mortality predictors							
PhenoAge	− 1.169	4.808	1.352	5.252	3.227	0.002	0.010
GrimAge	0.317	2.446	-0.274	2.132	1.663	0.098	0.140

### Comparisons of epigenetic age surrogate markers (cell count and GrimAge) between normotensive and PE

To further understand potential underlying differences in *DNAm*Age between women diagnosed with PE and those with normotensive pregnancies we also conducted independent t-tests for average estimated immune cell count measures and GrimAge *DNAm*-based plasma protein estimates. These results are presented in Table 4. After accounting for multiple testing (*p* < 0.007), two of the six (eosinophils were estimated at 0 for both groups, so not considered) estimated immune blood cell counts were significantly different between groups. B cells were found to be significantly lower (*p* < 0.002) in women with PE than those with normotensive pregnancies. Natural killer (NK) cell estimates were also found to be significantly lower (*p* < 0.001) in women with PE when compared to women with normotensive pregnancies. Of the eight GrimAge *DNAm*-based plasma protein estimates, two were significantly lower, *DNAm*adm (*p* < 0.005) and *DNAm*pai_1 (*p* < 0.001) in women with PE when compared to women with normotensive pregnancies.

**Table 4:** Independent t-tests between average DNAm-based cell count and estimators of plasma proteins among normotensive and preeclamptic women.

	Normotensive (*N* = 89)		Preeclamptic (*N* = 77)		t	*p*	Cohen’s D
	Mean	Std.Dev	Mean	Std.Dev			
**Cell count**							
Bcell	0.021	0.010	0.016	0.01	3.01	0.002	0.471
CD4T	0.0816	0.022	0.076	0.050	0.89	0.374	0.148
CD8T	0.028	0.022	0.0240	0.02	1.24	0.217	0.194
Eos	–	–	–	–			
Mono	0.083	0.017	0.077	0.021	2.01	0.046	0.317
Neu	0.748	0.049	0.777	0.090	− 2.54	0.012	0.412
NK	0.041	0.022	0.027	0.022	3.789	0.00002	0.589
**Plasma based estimators**							
DNAmGDF_15	614.906	154.225	621.059	93.668	− 0.315	0.753	0.050
DNAmB2M	1,357,350.202	99,289.073	1,337,098.909	90,476.932	1.375	0.171	< 0.01
DNAmCystatin_C	520,079.352	23,319.247	525,917.460	23,215.985	− 1.612	0.109	0.030
DNAmTIMP_1	30,839.098	896.438	30,810.191	820.949	0.217	0.829	< 0.01
DNAmadm	329.143	15.197	322.506	14.753	2.850	0.005	0.440
DNAmpai_1	16,398.178	1867.509	15,138.470	1963.188	4.217	< 0.001	0.690
DNAmleptin	13,168.183	1639.029	12,641.786	2107.368	1.776	0.078	0.030
DNAmPACKYRS	2.721	11.064	4.189	12.071	− 0.813	0.418	0.127

Growth differentiation factor 15 (GDF15), cbeta-2-microglobulin (B2M), cystatin C, ltissue inhibitor metalloproteinases 1 (TIMP1), adrenomedullin (ADM), plasminogen activation inhibitor 1 (PAI-1).

## Discussion

This study sought to determine if maternal *DNAm*Age and associated epigenetic age acceleration measures were significantly different in women with PE and those with who had normotensive pregnancies. Seven commonly utilised methods for calculating *DNAm*Age were considered: Horvath, Hannum, PhenoAge, Horvath Skin and Blood, Wu, GrimAge, and TL. We also estimated three epigenetic age acceleration measures: *ΔDNAm*Age, EAA, and IEAA as well as surrogate *DNAmAge* measures of immune cell count and plasma protein function. We confirmed that each of the seven investigated *DNAmAge* measures were significantly correlated with chronological age. Independent of covariates we observed a significant increase in *DNAmAge* in the Wu model^19^ between women with a normotensive pregnancy and those with PE. In addition, we also detected increased *ΔDNAm*Age, EAA, and IEAA for three *DNAm* measures (Wu, Hannum, and PhenoAge). Finally, we also found significant differences between four surrogate *DNAm* measures (Bcell, NK, *DNAm*adm, and *DNAm*pai_1) between PE and normotensive pregnancy. These findings suggest some estimates of *DNAmAge* are associated with differences between PE and normotensive pregnancies and highlight the possible role of differential DNA methylation in increased maternal susceptibility for PE complicated pregnancies.

Only three (Hannum, Wu, and PhenoAge) of the investigated measures of *DNAmAge* and EAA, demonstrated a significant difference between women with normotensive pregnancies and PE. This may be indicative of different underlying DNA methylation components being associated with *DNAmAge*. Two (Hannum, Wu) of these three measures identified as significant measure a subset of DNA methylation markers and were developed to correlate with chronological age rather than with disease. Also, these measures do not account for cell-type in their age estimations. The one previous study that investigated *DNAmAge* and PE^27^, did not identify an association between *DNAmAge* and PE among 56 pregnant women. However, the *DNAm*Age method they utilised incorporate cell counts into the model, potentially indicating that accounting for immune function in the statistical estimate may confound differences between normotensive pregnancies and PE.

There is a current trend for *DNAm*Age estimates to move toward composite epigenetic DNA methylation scores that are validated or enriched for disease morbidity and additional biological risk factors^37^. PhenoAge represents one of these measures and captures DNA methylation markers known to be optimized for mortality risk among individuals with the same chronological age^20^ whereas GrimAge incorporates chronological age as an adjustment variable in the model along with the additional plasma protein and estimated smoking variables to predict time to death. This may indicate why PhenoAge demonstrated the lowest correlation when compared the other *DNAm*Age measures due to the diluted contribution of chronological age in the prediction model. However, the PhenoAge estimates were considerably closer to the actual chronological age of the two groups of women (33.1 years for normotensive and 35.86 years for PE) than GrimAge, which provided the highest estimate for *DNAm*Age (39.51 years for normotensive 39.59 years for PE). This difference is further shown when investigating all three measures of epigenetic age acceleration, where PhenoAge was significant, but GrimAge was not. This may be representative of the different targeted CpGs in each of the models as there is often very little overlap of DNA methylation markers between the two models.

Advanced maternal age is known to lead to variety of pregnancy complications^38,39^ and birth defects^40^. However, we did not observe a statistically significant difference between chronological age and PE. This result is in contrast with two recent studies that have shown that women with advanced maternal age have increased risk of PE^25,26^. Both these studies found women with high advanced maternal age, > 35 year for Lamminpää et al. and > 45 years for Sheen et al., were at the highest risk for developing PE. In our case the average chronological ages for women with normotensive pregnancies (age = 31.76) and PE (age = 30.53) were similar. Among all *DNAm*Age estimates we measured the highest correlation with chronological age was observed with Horvath Skin and Blood *DNAm*Age (r > 0.7). Of the six estimated *DNAm*Age measures, Wu *DNAm*Age estimated the average age of normotensive pregnancies to be 9.92 years versus 10.33 years for women with PE, which is a severe underestimate of their chronological age. However, the Wu *DNAm*Age estimate weakly predicted chronological age (r = 0.265 among normotensive and r = 0.627 among PE pregnancies). Moreover, Wu et al.^19^ developed their *DNAm*Age estimate for investigating the prevalence of age-related diseases among younger individuals and several of the CpGs are associated with pregnancy or early life growth. This may have influenced the estimates within our study as the DNA samples from this cohort were collected at the end of pregnancy.

Due to discrepancies among investigated *DNAm*Age estimators, we also hypothesised that the differences in the results might be the effect of the number of included CpGs or the loci in which these CpGs are located that may be involved in the pathogenesis of PE. There is possible compromise in the prediction in the Wu estimator due to less CpGs in the model than in most of other estimators. The lower number of CpGs in the model might be due to the predicted age among younger individuals using a more restrictive model. Additionally, Wu’s model was validated for 67 pairs of monozygotic twins, whose genetic background and declared environmental exposure were very similar^19^.

The role of epigenetic age acceleration is known to be related to the age-related functional decline of the immune system and our results suggest this across three of *DNAm*Age models (Hannum, PhenoAge, and Wu). Accelerated epigenetic aging in the placenta as measured by Horvath *DNAm*Age or placental specific *DNAm*Age age has been associated with reduced birth weight and early-onset PE^24,41^. The placenta also can experience telomere length shortening that may induce cellular senescence that promotes parturition through increased inflammation^37,42,43^. In women with advanced maternal biological age these processes may be accelerated leading to pregnancy complications and poor birth outcomes. As we did not investigate placental tissue, additional research is required to determine the association between *DNAm*Age and different tissue types during pregnancy. Although not significant, our DNA methylation measures/analyses suggest that TL was lower among women with PE when compared to normotensive women.

Associations between the estimated cell counts B cells and NK cells as well as the GrimAge surrogates *DNAm*adm and *DNAm*pai_1 were consistent with previous literature in relation to pregnancy and PE. Auto-antibodies are produced during PE and have been previously shown to be higher in women with PE than in those who had normotensive pregnancies^44^. Natural killer cells play an important role in human pregnancy and regulation of these cells contribute to reproductive success and it has been previously shown that women with PE have a decreased percentage of NK cells when compared to those with a normotensive pregnancy^45^. Both *DNAm*adm and *DNAm*pai_1 were validated from biomarkers involved in hemodynamics^46^. Biological variation in hemodynamics risk are important during pregnancy to ensure proper circulation to the placenta and meet the increased metabolic demands of the developing foetus. Plasminogen activator inhibitor type 1 (PAI-1) represents a major down-regulator of fibrinolytic activity that results in the reduction of blood flow to the placenta which initiates the release of factors that activate maternal vascular endothelium resulting in PE^47^. This is supported across several studies that have demonstrated higher plasma levels of PAI-1 in women with PE compared with normotensive women^48–51^. Adrenomedullin (ADM) is a pro-angiogenic peptide hormone that regulates blood pressure and vascular integrity, is highly expressed in both the placenta and maternal vascular endothelial cells and has been shown to be associated with PE^52^. This demonstrates that the use of DNA methylation estimate as a potential biomarker for PE can be done successfully and be representative of the immune function and hemodynamic process in pregnancy. However, this should be interpreted with caution as these are estimates drawn from whole peripheral blood samples and these DNA methylation measures were not correlated with measured blood derived markers.

There is evidence that global DNA methylation may influence PE risk during the placentation process^53^ and is related to maternal blood pressure^54^. DNA methylation data applied in epigenetic age calculations are also linked with the gestational age of the placenta which is positively correlated with pregnancy complications such as PE^24^.

This current study is limited by its observational nature, whereby direct causation cannot be ascribed, but only inferred. The study was also not adjusted for additional confounders such as socioeconomic status, dietary habits, medication, or stress level during pregnancy, preventing any investigation how these variables may impact the association between *DNAm*Age and PE. A further limitation is that DNA methylation was measured from whole-blood samples. This introduces complexity in interpretation of the contribution of cellular heterogeneity, which we controlled for through well-established methods for estimation. We have not incorporated epigenetic estimates for mononuclear leukocytes, which include macrophages and dendritic cells, that migrated out of the blood stream into tissues. The estimation of these mononuclear leukocytes from CpGs may be possible from specific tissue types, such as the placenta, but not from whole blood as used in this research. An additional limitation of the study is that biological samples for the participants were collected at the end of their respective pregnancies and did not incorporate information about time of PE onset (early or late onset PE). However, we hope that our current manuscript prompts other researchers in the fields of obstetrics and epigenetic ageing to further investigate this topic.

Finally, the wider relevance of these observed *DNAm*Age estimates require replication in other pregnancy cohorts.

## Conclusions

This study represents the first study to systematically evaluate the association between maternal *DNAm*Age and PE across both chronological age and mortality predictors of epigenetic age. We find that accelerated maternal *DNAm*Age is associated with PE in three different models of *DNAm*Age (Hannum, PhenoAge, and Wu). These findings underline the importance of the potential DNA methylation modifications that differentiate PE and normotensive pregnancies. The application of composite genomic risk scores, such as genetic risk scores or *DNAm*Age have been recently developed in the research setting for their potential use in the clinical setting for primary and secondary prevention^55,56^. To date, their potential application has been proposed for several complex diseases including cardiovascular disease and cancer, but the role of epigenetic aging during pregnancy is less clear.

Further investigation would be beneficial to better understand how epigenetic age acceleration interacts with environmental factors and fixed effects in PE to determine how modifiable these *DNAm*Age estimates are during pregnancy.
